# Camphor—Poisoned by

**Published:** 1861-09

**Authors:** 


*Camphor (Poisoning by).*—Dr. Fenerly relates (Journal de Chimie Medicale, June, 1860), the case of a woman, aged 36, in the fourth month of pregnancy, who took twelve grains of camphor to produce abortion. For two hours she was intoxicated, had headache, redness of face, burning sensation in the stomach. After eight hours, increasing pain in the epigastrium, extending to the loins, diffused also through the whole abdomen; stranguary, excessive anxiety, vomiting. Three hours later, face pale, bluish, eyes hollow, skin cold, insensitive, pulse thready, heart's impulse weak and slow, respiration difficult, voice weak, abdomen intensely painful to the touch, violent spasms of the extremities, micturition suppressed for twenty-four hours, bladder excessively full. Slight discharge of blood from the vagina; os uteri widened, very hot. The patient lived three days longer, and aborted on the evening before her death. No autopsy.—*American Medical Monthly.*

